# A Journey From Cardiology to Oncology Reveals a Rare Case of Primary Intimal Sarcoma in a Patient With Dyspnea: A Case Report

**DOI:** 10.7759/cureus.38439

**Published:** 2023-05-02

**Authors:** Ahmad Mahdi, Mahmoud Mahdi, Patrick Ters

**Affiliations:** 1 Internal Medicine, University of Kansas School of Medicine, Wichita, USA; 2 Cardiology, University of Kansas School of Medicine, Wichita, USA

**Keywords:** dyspnea, interventional cardiology, oncology, case report, primary intimal sarcoma of the heart

## Abstract

Primary intimal sarcoma of the pulmonary artery is a rare and aggressive malignancy that arises from the intimal layer of the pulmonary artery. It typically presents with nonspecific symptoms such as dyspnea, chest pain, and hemoptysis, making early diagnosis challenging. Computed tomography (CT) and magnetic resonance imaging (MRI) are useful in identifying the tumor's location and extent. A definitive diagnosis is established by biopsy, either via surgical resection or percutaneous needle biopsy. However, diagnosis can be difficult due to the rarity of the disease and the need for specialized expertise in interpreting pathology specimens. Treatment of primary intimal sarcoma of the pulmonary artery involves surgical resection, followed by adjuvant chemotherapy and radiation therapy. Despite aggressive treatment, the prognosis remains poor, with a median survival of approximately two years. However, early detection and aggressive multimodal therapy can improve outcomes. We hereby report a rare case of primary intimal sarcoma of the pulmonary artery and discuss its pathophysiology, presentation, diagnostic approach, and treatment options.

## Introduction

Primary intimal sarcoma of the pulmonary artery is an extremely rare malignant mesenchymal tumor, with around 400 cases only published in the literature [1,2]. It originates from the intimal layer of the vascular wall, grows to obstruct the lumen, and invades the vascular wall, and fragments of the tumor may potentially detach and metastasize to distant organs along the downstream circulation. Its presentation is often falsely attributed to other more common pulmonary vascular diseases such as chronic pulmonary thromboembolism (CPTE) [3]. In this case report, we present a patient who presented with shortness of breath to an outpatient clinic and had an extensive workup, which included an excisional biopsy confirming the diagnosis of primary intimal sarcoma.

## Case presentation

An 81-year-old male patient with a medical history of coronary artery disease, having undergone a percutaneous intervention (PCI) in the past, and with a history of melanoma of the left shoulder (T3b, N0), visited his primary care physician due to progressive shortness of breath experienced for several months. He denied having any other related symptoms. During the physical examination, all results were normal except for the detection of a mid-systolic murmur of grade 3/6, which was heard at the left parasternal area.

**Figure 1:**
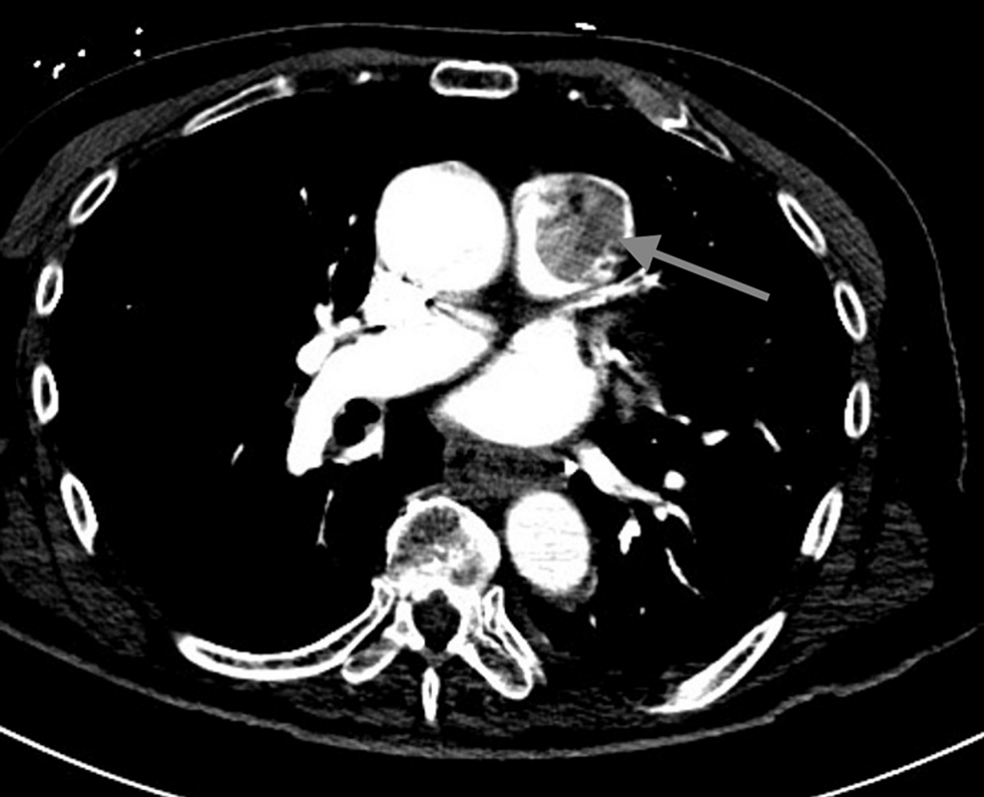
Transverse view CTA of the chest revealed a filling defect in the main pulmonary artery just above the pulmonary valve. CTA, computed tomography angiography

**Figure 2:**
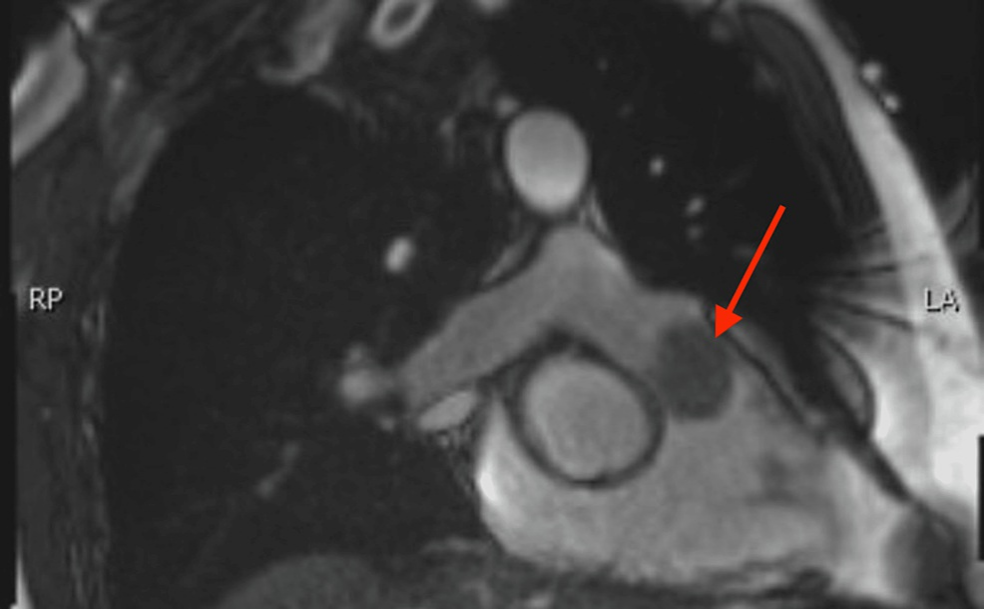
Functional cardiac MRI showing a mass attached to the pulmonic valve. MRI, magnetic resonance imaging

After being referred to a cardiology consult service, the patient underwent transthoracic echocardiography, which revealed significant insufficiency and stenosis of the pulmonary valve, and a very large echogenic structure measuring 2.8 cm × 3.2 cm. The structure was highly mobile within the valve and able to move freely through the right ventricular outflow tract (RVOT) system, raising concerns of an infection, although this was considered unlikely. Another possibility was a primary or metastatic tumor. A computed tomography angiography (CTA) of the chest was performed, which showed a filling defect in the main pulmonary artery just above the pulmonary valve (Figure 1). Further investigation included functional cardiac MRI with gadolinium contrast, which revealed a mass attached to the pulmonic valve, as well as additional nodular masses in the main pulmonary artery and expansion of the apical-posterior segmental artery in the left upper lobe with soft tissue (Figure 2). The MRI enhancement was observed only on the pulmonic mass, which had a component of the thrombus attached. Bacterial and fungal studies, including negative serologies for Bartonella, Brucella, Coxiella, Rocky Mountain spotted fever (RMSF), and Histoplasma, were negative.

**Figure 3:**
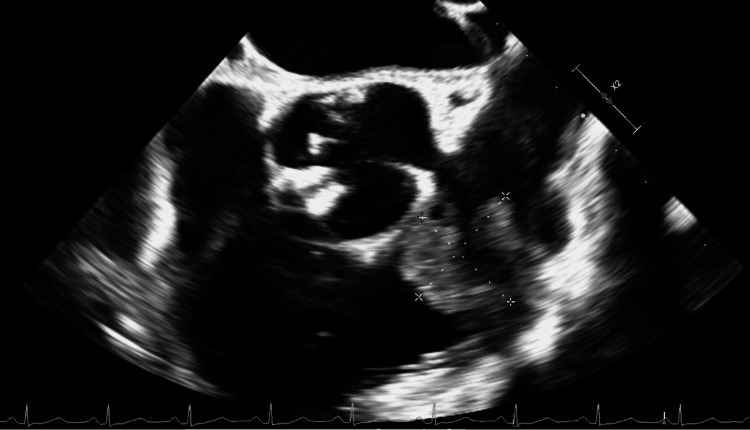
Transesophageal echocardiogram confirming the presence of the mass on the mid-esophageal aortic valve short axis view.

The cardiothoracic surgery, interventional cardiology, and oncology teams collaborated to determine that percutaneous biopsy would be unsafe due to potential complications such as uncontrolled bleeding, distal embolization, and cardiopulmonary arrest. A transesophageal echocardiogram confirmed previous imaging findings (Figure 3). The patient underwent successful surgical resection of the mass, and reconstruction of the main pulmonary artery, pulmonary valve, and RVOT. Immunohistochemical studies on the specimen were negative for block B1, BRAF, SOX10, smooth muscle actin, CD31, and caldesmon. Furthermore, the provided immunostains indicated that the neoplastic cells were negative for desmin, S100, MART1, and AE1/AE3. Genetic studies revealed an amplification of the *MDM2* gene region, which is identified in atypical lipomatous tumors, well-differentiated liposarcoma, and dedifferentiated liposarcoma. However, revision of the pathology slides confirmed the diagnosis of MDM2-amplified high-grade spindle cell sarcoma, consistent with pulmonary artery intimal sarcoma.

The rest of the patient's hospital course was uneventful. The patient was discharged from the hospital and lost for outpatient follow-up to resume his treatment plan, unfortunately.

## Discussion

Intimal sarcoma of the pulmonary artery is an extremely rare and strongly invasive malignant mesenchymal tumor that arises almost exclusively from the pulmonary artery wall in the pulmonary circulation [4]. Mutations in the *CDK4* gene are attributed to its pathogenesis, although the etiology remains unclear [5]. It occurs more often in females with an average disease onset of 50 years [6,7]. Since the malignancy resembles CPTE, the incidence rate is fairly underestimated [8]. It involves the proximal side of blood vessels, often in the pulmonary trunk (80%), left or right pulmonary artery (50%-70%), or both (40%). Portions of the tumor usually detach from the vascular wall and metastasize to remote organs, including lymph nodes, kidneys, lungs, brain, and skin [9].

Patients with sarcoma of the pulmonary artery remain asymptomatic until the mass obstructs the vascular lumen or results in stenosis of the pulmonary valve. Symptoms are often nonspecific and include shortness of breath, fatigue, cough, syncope, fatigue, chest/back pain, and symptoms of right-sided heart failure [10,11]. As the presentation is nonspecific, diagnosis becomes challenging [8]. Diagnostic laboratory workup is nonspecific. Electrocardiogram can reveal right ventricular hypertrophy secondary to pressure overload. Color Doppler echocardiogram reveals tricuspid valve regurgitation, right ventricular enlargement, and signs of pulmonary hypertension secondary to vascular obstruction [8]. In the case of our patient, the transthoracic echocardiogram (TTE) was able to detect pulmonary valve insufficiency and, luckily, the presence of the mass, although it is often very challenging, given acoustic windows, chest wall limitations, and the inability to see the pulmonary artery on TTE. CT pulmonary angiography (CTPA) is the imaging modality of choice for diagnosing pulmonary artery tumors. It reveals it as a mass invading the vascular wall or nearby structures or as a mass involving the pulmonary valve or the RVOT [12]. The golden standard, however, remains postmortem or postoperative histopathological examination of the mass as explained in the earlier case presentation [13].

In general, intimal sarcomas that affect the pulmonary artery tend to display histological characteristics that are consistent with undifferentiated or unclassified sarcoma. This is similar to our case and is usually a diagnosis reached by a process of elimination because specific soft tissues can often be identified through the use of immunohistochemistry and/or molecular genetic techniques in the majority of cases. In rare instances, these sarcomas may also feature areas that exhibit rhabdo-, osteo-, chondro-, or angiosarcomatous traits. Primary intimal sarcoma can be characterized by positive staining for vimentin, smooth muscle actin (SMA), desmin, CD31, and CD34. Vimentin and SMA are markers of mesenchymal differentiation, while desmin is a marker of smooth muscle differentiation. CD31 and CD34 are endothelial markers, and their expression in primary intimal sarcoma supports the theory that these tumors arise from endothelial cells. Other markers that may be expressed in primary intimal sarcoma include WT-1, ERG, and FLI-1. These markers are not specific to primary intimal sarcoma but may help to differentiate it from other vascular tumors [14]. 

The median overall survival of patients with malignancy is around 17 months [12]. Similar to our patient's case, surgical treatment is preferred and can prolong the survival period [7]. Chemotherapy, on the other hand, is often used postoperatively or is reserved for non-operable or relapsing tumors [15]. Several agents are reported, including anthracyclines, gemcitabine, ifosfamide, platinum, taxanes, and immunotherapy. Anthracyclines alone or in combination are most commonly utilized [12]. Chemotherapy is often followed by intense modulated radiation therapy (IMRT) [16]. Otherwise, the only targeted therapy for soft tissue sarcoma, including pulmonary primary intimal sarcoma, is tyrosine kinase inhibitor pazopanib according to the PALETTE trial [17].

## Conclusions

Intimal sarcoma of the pulmonary artery is an extremely rare and invasive malignancy with a nonspecific presentation. In addition to high clinical suspicion, imaging modalities, such as CTPA, can be used for diagnosis. Surgical resection is the gold standard for treatment and can prolong life expectancy.
